# The ART and LOGIC of scholarly communication: Effective skills for publication and beyond

**DOI:** 10.1002/fsn3.2766

**Published:** 2022-04-13

**Authors:** Yangming Martin Lo

**Affiliations:** ^1^ 47890 Institute for Advanced Study Shenzhen University Shenzhen China

## Abstract

Based on a series of well‐recognized workshops around the world conducted by the author with more than 15 years of EIC experience, this article highlights the essence and spirit of publishing scholarly outcomes for authors in science and engineering. It is vital for all scientists who intend to upgrade their publications to bear in mind that, additional to KISS (Keep It Simple and Straightforward), the contents of a manuscript should be prepared based on the core concept of ART and LOGIC, namely:

- Thoroughness (be thorough when compiling)
  o Complete the sentence, paragraph, and article
- Readability (keep the expression readable)
  o Know what you mean and mean what you know
- Articulateness (be articulate in wording)
  o Be polite yet firm, when necessary, with controversies

and

- Lock one in ASAP
  o Identify and be identical with the readers
- Offer useful information
  o Justification and reasoning are most critical
- Gain confidence
  o Put forth best methodology and know‐how
- Indication of good will
  o Be constructive even when criticism is needed
- Conclusive statement
  o Provide solid conclusion with proper outlook

## 1 INTRODUCTION

Publication in peer‐reviewed journals or so‐called SCI (Science Citation Index) journals has been adopted in most countries as the upmost and in many cases the only deciding criteria in assessing accomplishments of scholars, from annual performance review to career‐hinging promotions and tenure evaluations. In some countries, the SCI impact factor alongside the order of authorship (first or corresponding author) is quantified and tied with annual bonus and monetary rewards. Therefore, the amount of manuscripts generated keeps growing exponentially as the phrase “publish or perish” attests to be more valid than ever. However, as the number of journals continues to grow exponentially, it becomes apparent that the quality of scientific papers yields a much wider discrepancy, and the job for editors and peer reviewers becomes more difficult through having to deal with manuscripts carrying significant frauds.

In science, in the author’s humble opinion, there are two types of scientists. The first is the ones who are fearless and creative enough to lead us to an unexplored territory, showing the world of untapped frontiers. There have been few to date that could be appraised into this category. The majority of scientists build their research on the foundations established by predecessors. The potent ones contribute like adding bricks onto a wall of newly poured concrete for a house, and the rest manage to put on wallpaper or decorative lamps in a house that is already been built. How do we differentiate the value of a scholarly finding? It is critical to recognize one simple word K.I.D., where D stands for data, I for information, and K for knowledge. The daily experimental outcomes acquired by relentless graduate students and research associates are considered merely “data” in the scientific world. Data remain as data in one’s collection if it is not verified by other experts in the same field. Once a research group accumulates sufficient data that could indicate a trend or explanation for certain phenomenon, it is the time for the group to put together a manuscript, which is intended to share the data in a meaningful sense for peers to assess its merit. Manuscripts, at this stage, are viewed as precursors of “information” worthy of circulation. The manuscripts that have undergone peer review and considered meritorious for publication become “information” once it has been corrected, typeset, and carries an issue and page number of a reputable journal.

Before the “information” can be published, it is important to have a clear idea about authorship here. In some countries, the requirement of having several “first‐authored” articles is built‐in together with needing to have served as the “corresponding author” for a number of articles before one can be granted a graduate or Ph.D. degree. The FIRST author is the one who provides the most contributions to the work, either by having the most critical piece of knowledge that resulted in the scientific breakthrough, or the one who conducted the most amount of work that shaped the findings included in the manuscript. The order of authorship ascends accordingly. The CORRESPONDING author, on the contrary, should be the “owner” of the research group, namely the academic director or the advisor of the research who could effectively answer any questions during and post review. In many cases, the first author is the graduate student whose degree thesis or dissertation forms the majority, if not all, of the manuscript, and he/she graduates around or after the paper is published. Such a student should never serve as a corresponding author because he/she will not be able to correspond to any challenges about the research outcomes down the road. The research director who retains the lab notebooks and builds the laboratory around the research topics is ideal to serve as the corresponding author, since he/she will be able to accept skepticisms or challenges by advising more research to prove or validate the findings published.

The “information” published in various journals and proceedings is circulated and assessable throughout the world for researchers to view, analyze, digest, and build new hypothesis upon. Therefore, while authoritative journals continue to try their best to upkeep the quality and ethics of the articles during the review and publication process, it is the responsibilities of the authors to ensure the data, the discussion, and in some cases the postulation, adhere to the facts and findings. It should be treated almost religiously for each researcher to keep a lab notebook detailing every single piece of their daily progress and findings. The notebook should then be reviewed by their labmates or supervisor/advisor periodically and signed, dated to ensure the originality of the data. While digital lab notebooks might be acceptable in some fields of study, it remains a common practice for many to keep hard copies of the data in the notebook. The notebooks are a deciding evidence when it comes to awarding Nobel Prizes or other renowned awards because these awards demand traceability back to the time when the idea or evidence was originally created.

One day, after many rounds of scrutinization and challenges, as well as applications that might derive out of it, the “information” created by you becomes golden rules such as E = mc^2^; then, it evolves into the highest level “Knowledge,” the K of K.I.D.

## 2 PREPARATION OF A MANUSCRIPT

So, now you have completed all the experiments under a valid hypothesis and found something interesting and worthy of publication after discussion with your advisor/supervisor. How do you start preparing for the manuscript? In general, a regular scientific paper consists of Abstract, Introduction, Materials and Methods (M&M), Results and Discussion (R&D, together or separately), and Conclusion. Which part should you begin with first? It is highly recommended to start working on the details of Materials and Methods, since you are the one most familiar with the experimental design and all parts of tasks involved. It is also a good feeling when you can write beyond page one upon first attempt. The most frustrating experience would be to dwell on page one over and over again without making progress that could impress yourself.

## 3 WORKING ON “MATERIALS AND METHODS”

Remember, there is no “you, he/she, and I” in M&M. The most important info delivered here should be the most updated, state‐of‐the‐art technologies adequate for the research, unless justifiable otherwise. The sequence of presenting M&M should be somewhat in sync with R&D. In many cases where the authors are caught with a high percentage of duplications or even plagiarism of another published paper, it is due mainly to a copy‐and‐paste of the same methodology from another paper. It is more critical nowadays than before as most journals use digital services such as iThenticate that could easily pick out text duplications. Articles with a high percentage of duplicated text often get stopped upfront and do not even reach the editor and/or reviewers. Please remember to cite the methodologies precisely, even if the previous work was conducted by the same group or same authors. Moreover, when writing M&M, it is essential to use past tense for the procedures because all the tasks “were” conducted by you. Never write it as a user’s manual or an experimental protocol you probably acquired from a previous graduate student or labmate. Carefully check through every step involved in the experiments, and use the correct measurement units, especially when weight by volume (w/v) or weight by weight (w/w) makes a difference. Proper statistical design needs to be in place from the very beginning of your experiments, with special attention on proper sample size and experimental repetition employed, although it is customary when writing M&M to place statistical design at the end of the paragraph/section.

## 4 WORKING ON “RESULTS AND DISCUSSION”

After completing the draft of M&M, it is a normal flow to begin working on R&D. Rule number 1 when writing R&D is that you should always begin with your Results first, then Discussion. Since the results were conducted by you, so only past tense should be used to describe your results. This is the heart and soul of your manuscript, and it is of the utmost importance to make sure your results are presented in the most logical/meaningful way, let it be a figure or a table. Also, the flow of contents needs to be kept as seamless as possible, so do not let languages such as Figure 1 shows ABC or Table 1 explains EFG get in your way. It is advisable to place references to the corresponding figures and/or tables in parenthesis instead.

How do you decide whether a figure or a table is most suitable for your data? It is preferable, in general, to use a figure if a trend or a relatively straightforward visual comparison is to be shown. The dots in the same figure should only be connected if the parameter is generally recognized as a “continuous” parameter, for example, temperature, time, or pH. However, cautions should be exercised on parameters such as pH because the ionic strength in the solution might cause an unexpected dip or spike in the value. In another word, it is the responsibility of the authors to make sure there is no surprise when making a smooth connection or regression curve between two dots in the figure. Equally noteworthy is the inclusion of error bars to indicate statistical significance whenever possible.

When complicated results are in need of comparison across a variety or a wide spectrum of parameters, especially when these parameters are independent or distinctly different from one another, a table might serve the purpose better. Again, proper care should be given as to the significant digits used when measuring each of the parameters, as well as how the statistical significance should be labeled with corresponding superscripts. All captions for figures and tables need to be able to tell a stand‐alone story of the data presented in the respective figure or table with detailed description of its statistics, so the readers do not have to dive into the texts to look for details.

Upon constructing Discussion, it is crucial to collect all relevant literature and discuss them following the sequence of “compare” then “contrast.” Compare, which follows immediately after your results, means citing the literatures that showed similar trends or in support of your findings, so you could iron out the improvements you have made in your research or link your findings with possible underlining theories or mechanisms. Contrast, on the other hand, means how your data might differ or oppose what have been published. It is important not to overlook any literature that is in direct conflict with your findings. This is the place where you can find justifications for your data, while pointing out the rationales attributed to the discrepancy between the literature and your work. Most manuscripts get rejected during the review process simply because the authors fail to address prior knowledge contradicting the results.

## 5 WORKING ON “INTRODUCTION”

While it might feel strange or at least somewhat awkward to work on the Introduction of your manuscript so late in the writing process, it is crucial that the “Introduction” section serves its purpose to justify the value of your work. In a nutshell, one could view the Introduction section as the “GPS” (Global Positioning System) of your work, that is, to clearly indicate and/or locate where your contribution is in the huge ocean of scientific literature.

The introduction section is where you plug in the aforementioned LOGIC guideline. The scope and relevance of your work should be clearly identified during the first two sentences of your first paragraph in order to “lock the readers in ASAP.” It is also critical to develop a holistic statement to break open the first paragraph so that it is not like, oh, A had said this, B also said this, so now I am saying the same thing here. It should definitely be a “synthesized” statement that the readers could immediately see your expertise in analyzing existing knowledge or information to come to a valid, take‐home‐possible opening statement.

Once the tone is set, you can then supply evidence to supplement or support your opening statement. Let it be positively supportive or even quasi‐negative or totally opposite viewpoints, it is your duty as the author to lay them all out here. The key is to present them in a logical manner, for example, organize them in a way that the readers could see how you group the information and how they relate to each other. This should go no more than two paragraphs at most and could be carefully arranged with proper citation of relevant references.

Then, roughly into the third or fourth paragraph, it is the time for you to narrow down the scope to the “weakest link,” namely what the void is in the literature or where previous literature might have fallen short, or it could be a clear indication of a dire need that had hindered the development of certain knowledge or technology. This is the place where you address carefully how your study is critical and crucial to contribute to the breakthrough, and should be cohesive to both the scope and the actual contexts of your findings.

The Introduction section could then end in a description of what your goal and objectives are, and at times maybe also an indication on what types of study you intend to perform to certain extent. This is also a good place to make proper linkage with your Materials and Methods.

## 6 WORKING ON “CONCLUSION”

One can only conclude what you found in the study, period. While you might feel there are many valuable points worthy of showcasing once more, here is not the place to do it. Keep the conclusion strictly adherent to your own findings as reported in the article, and nothing more. Do not try to cite any references in the conclusion, as they should have been cited and properly discussed in the main text already. Once you are happy with your conclusion, you will want to take a look at your article Title and Introduction altogether. Not only should the three parts echo each other, but they should also remain cohesive and relevant.

## 7 SUMMARY

Publication is not the end of science. It is just a beginning. One good solid piece will enjoy years of citations and discussions, and it could also lead to invitations for scientific seminars or keynote speeches at significant scholarly events. It is the building block of your academic and/or scholarly reputation. I hope this article could provide you some guidelines and considerations toward developing a successful career in science and technology.

